# Lessons learned from the COVID-19 pandemic—what Occupational Safety and Health can bring to Public Health

**DOI:** 10.1057/s41271-023-00394-w

**Published:** 2023-01-16

**Authors:** Lode Godderis, Loic Lerouge, Yogindra Samant, Peter Noone

**Affiliations:** 1 Centre for Environment and Health, and IDEWE, Occupational, Medicine, University of Leuven, Leuven, Belgium; 2 Labour and Occupational Health Law, University of Bordeaux-CNRS, Talence Cedex, France; 3 Norwegian Labour Inspection Authority, Trondheim, Norway; 4 Health Service Executive Dublin North East, Meath, Ireland; 5 KU Leuven, Environment and Health, O&N5b, Herestraat 49, Bus 952, 3000 Leuven, Belgium

**Keywords:** Prevention, Public health, Occupational health, Policy, Collaboration

## Abstract

We strive to increase public (PH) and occupational health (OSH) inter-linkages by building a collaborative framework. Besides Covid-19 pandemic, recent approaches such as Human Exposome and Total Worker Health TM, have led to a shift to improving health of working population and consequently the total population. These health objectives can be best realised through primary care actors in specific contexts. Work, school, home and leisure are the four multi-stakeholder contexts in which health and healthcare (goal-oriented care) objectives needs to be set and defined. PH policy makers need to establish a shared decision-making process involving employees, employers and OSH representatives to set PH goals and align with OSH goals. The policy making process in OSH can serve as a potential way forward, as the decisions and policies are being decided centrally in consultation with social partners and governments. This process can then be mirrored on company level to adopt and implement.

## Key messages

- Synergies and close collaboration between  occupational and public health professionals are key to prevent community-acquired infections, as has been demonstrated during the COVID-19 pandemic.
- Work, school, home and leisure are four multi-stakeholder contexts in which we propose public health professionals to define and set both health and healthcare (goal-oriented care) objectives.
- Public Health objectives are best realized locally both with and within the local community in conjunction with relevant OSH stakeholders.

## Introduction

The COVID-19 pandemic has raged for more than two years now. With the succession of new variants, it appears that it will remain pandemic for the foreseeable future. Many European countries are gradually relaxing pandemic restrictions, but we need to remain vigilant. The impact of the pandemic in terms of the cost to human lives, health, and socioeconomic stability of society has been very big [1]. This Viewpoint focuses on how Occupational Safety and Health (OSH) can leverage optimal impact on public health (PH) through closer collaboration, based on our experience in Europe. We advocate stronger links between public and occupational health with a clear structure to define this cooperation to achieve better synergies and outcomes.

Before the pandemic, European countries paid little attention to employers as critical actors in prevention to protect the health of the population. At the turn of the nineteenth century, PH and OSH emerged from common origins in Europe, but the role of public health in this region has been more limited than in the United States (US), which are partly due to differences in level of unionization and collective bargaining culture. Hence, European OSH policy operates in a tripartite framework involving employers, workers, and, in most countries, ministries of labour or labour inspection (OSHA). Labour unions in many European countries have been mainly focussing on work-related diseases and injuries keeping out of the OSH and tripartite framework lifestyle related PH outcomes that may have a workplace attribution like obesity, cardiovascular diseases, and diabetes.

Europeans have treated workplace health promotion and infection control as mostly within the domain of public health, with, at best, the involvement of the tripartite framework. For example effective tuberculosis control and prevention is done in an integrated program with all general health care providers and stakeholders—including government health facilities, nongovernmental organizations, private and OSH practitioners, community groups, and workplace actors. The pandemic has thrust infection control and perhaps also health promotion (home office, sitting, and screen time) into the workplace domain. Thus, it is likely employers will become more involved in PH prevention along with the employees and labour inspectors and ministries.

COVID-19 has highlighted the employer's role in preventing occupational and other PH risks in the workplace in close collaboration with the OSH practitioners. OSH practitioners participated in the PH pandemic response in various ways and degrees of efficiency and effectiveness. A first, OSH providers undertook actions at an individual level: testing, tracking, isolating, and vaccinating workers in occupational settings. They also performed workplace risk assessments and advised employers about implementing exposure controls such as source elimination, containment, segregation, pathway controls (effective ventilation and filtration) and receptor controls (effective personal protective equipment (PPE)) [2, 3].

OSH practitioners also played important roles in prevention and mitigation of physical and mental health problems at collective and individual levels [4, 5]. OSH provided advice on ergonomic and psychosocial issues for healthcare workers and people working from home [6]. These activities demonstrate importance of OSH important input with employers and for policy to ensure that working people could be appropriately protected and critical services and economic activity could continue uninterrupted [7].

Workplaces afford contact of staff with the general public, customers, suppliers, visitors, and patients. Because employees interact, cohabit, commute to work together, they are potential carriers, vectors, or targets of the virus. Effective workplace prevention can reduce excess occupational risk for workers, their families, community contacts, and patients in healthcare [8–11]. Workplaces also serve as settings for education about prevention and vaccination, knowledge that workers can transfer to their communities. Thus, OSH contributes to effective prevention of community-acquired infection.

Conceivably the lack of integration between OSH and PH pandemic early response lead initially to the neglection of necessary precautions (e.g. provision of appropriate Respiratory Protective Equipment) and occupational risk assessments to protect both staff and business, which facilitated wider transmission driving natural selection of SARS-CoV-2. Effects include increased transmissibility (seen with alpha, delta, and omicron variant), increased immune escape (beta and omicron variant), or greater pathogenicity (alpha and delta variants). This continues with variants BQ.1.1, BF.7, BA.2.75.2, BA.2.3.20 and XBB amongst others. Countries that permitted high transmission have had higher COVID-specific and all-cause mortality, healthcare worker shortages, and repeated lockdowns to control surges in cases [12]. Countries that suppressed transmission early reduced mortality and have, to date, suffered less economic damage. European responses to COVID-19 have highlighted the value of an integrated approach, also called the Swiss Cheese Model.

**Fig. 1:**
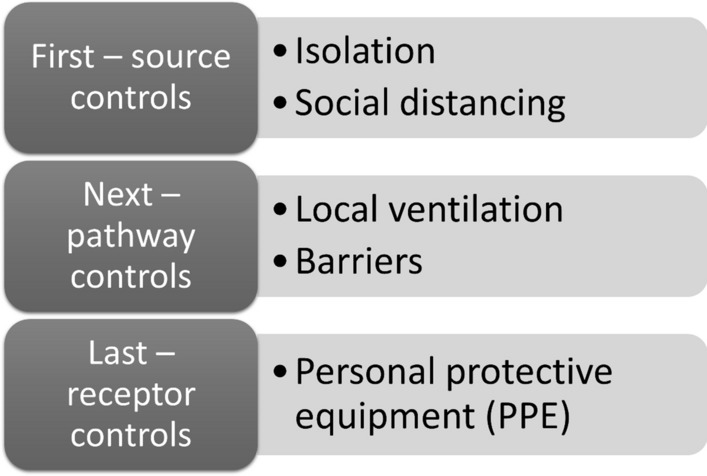
Paradigm of integrated control measures

This model is also central to management of other crises affecting communities. A ‘vaccines-plus' strategy exemplifies the sort of integration of OSH and PH that we advocate. As depicted in Fig. 1, this strategy combines vaccination, exposure control, and financial support. For exposure control, intervening at the source control is most effective; receptor control is the least effective measure [13]. If countries worldwide were to integrate and apply all these measures, they could reduce the evolution of new variants by ensuring low transmission. Effective PH measures can contain outbreaks, while ensuring that everyone (including the clinically vulnerable) can live and work freely.

To be more effective in controlling the pandemic, we argue that PH and OSH must work together in a complementary fashion. Several EU policies, documents, debates, and research programs highlight movement in this direction since inception of the pandemic [14]. This alliance is critical to increase the preparedness for potential future health crises, as outlined in the EU Strategic Framework on Health and Safety at Work 2021–2027 [15]. The European Commission advocates that “synergies between OSH and PH should be further developed”. An in-depth assessment of the effects of the pandemic and the efficiency of the European Union (EU) and national OSH frameworks will be necessary to develop emergency procedures and guidance for the rapid deployment, implementation, and monitoring of effective measures in potential future health crises, in close cooperation with PH actors. The Commission should move rapidly to determine how to achieve such ‘synergies’ and to promote them across Europe. Only recently EU has taken action and put the building blocks of the European Health Union officially in place. This not only includes stronger EU rules on serious cross-border threats to health, but also a stronger mandate of the European Centre for Disease Prevention and Control (ECDC) and a new Emergency Framework for medical countermeasures.

## Synergies between OSH and Public Health

In recent decades, PH and OSH scientists have been working to improve the health of the working population, as this contributes to improving the health of entire populations. An illustration in the field of PH is the human exposome approach. It aims to characterize an individual's exposures from the cradle to the grave, including diet, air pollution including occupational exposure to chemical, physical, biological, and psychosocial hazards [16]. The holistic approach of the exposome requires consideration of the occupational and public health impacts of the processes of an enterprise. This approach shifts the focus to prevention and must include health promotion and ill-health prevention in the workplace.

The Total Worker Health™ approach from the United States National Institute for Occupational Safety & Health (NIOSH) created a model for OSH. The goal is to create a hazard-free work environment protecting all workers. It brings together positive aspects of work workplace interventions that contribute to health, including health promotion and return-to-work programs [17].

The pandemic eroded boundaries between work and personal life, enabled by digital technology, creating a continuum of work from ‘boardrooms to bedrooms’. An increasing number of people have been working from home, providing both benefits (no commuting time, picking up children from school) and challenges including difficulties to disconnect from work or securing adequate work-life balance. The rapid evolution of home-based working during the pandemic highlighted employers' obligations to create healthy and safe workplaces in private homes. Courts in several EU countries have increasingly recognized accidents of teleworkers at home as occupational accidents, meaning they place responsibility on employers. In France, Civil Law, Art. L. 1222-9 of the French Labour Code created a presumption that an accident occurring during telework is an occupational accident [18]. In Germany the Federal Social Court (“Bundessozialgericht”) recognized as an occupational accident that of a plaintiff who suffered an injury when he fell while walking to his home office in the morning.

In this boundaryless work life interface privacy of the individual needs to be respected. An employer might gain digital access to the workplace inside private homes to identify and address hazards. Such access, however, could facilitate unwarranted and sustained digital surveillance to monitor employee productivity or performance. Public health authorities need to address the entire population and acknowledge OSH expertise for controlling occupational exposures and addressing issues specific to workplaces. Public and occupational health practice share common aims and apply similar methods:

- Health improvement, including surveillance, monitoring, and assessing epidemiological risk factors and disease prevention.
- Health protection, through reduction of exposure.
- Healthcare, including service planning, access, and evaluation.
- Reducing inequalities in health.

## Realising health objectives through primary care actors working in specific contexts

PH is the science and art of preventing disease, prolonging life and promoting health through the organised efforts of society. OSH focuses on all those who work, and OSH combines individual and population-based approaches, connecting the workplace with all other dimensions of the workers’ health.

**Fig. 2:**
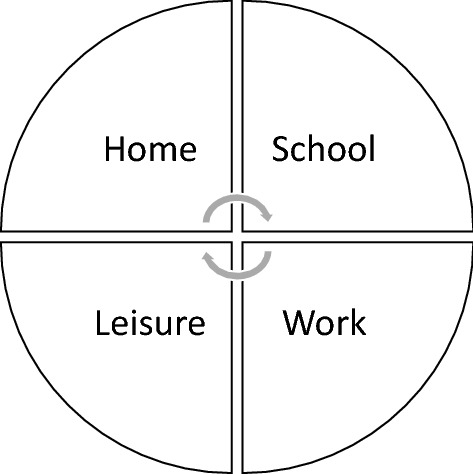
An integrated policy and regulatory framework for occupational and public health

The 1978 Alma Ata declaration called for a shift in focus from reactive management of sick individuals to prevention and health promotion at the community level [19]. Work, school, home, and leisure (Fig. 2) are the four contexts in which we propose PH to define and set objectives for health and healthcare (goal-oriented care). PH must link all the relevant actors to collaborate and focus on each person as well as the entire population.

Hence, we advocate that PH work to achieve its objectives locally with and within the local community in conjunction with OSH stakeholders. PH authorities must finance local actors and align incentives for achieving population level prevention. The policy making process in OSH is a good model for how to make decisions and policies centrally in consultation with social partners and governments. Occupational medicine and General Practitioners (GPs) should mirror this process to develop and locally implement OSH policy within companies. They can also contribute to data collection and analysis. These recommendations align with the 1978 Alma Ata declaration for prevention and health promotion at community level [20].

To improve outcomes, the model calls for PH agencies and authorities to include the workplace environment as an important contributing factor in population health data along with individual behaviours such as smoking, alcohol abuse, or unhealthy diets [21]. PH will need to consider all workplace influences on all diseases and inequalities determinants for the health of the entire population. Cancer registries will need to include proximate and distant work history of all those registered to get a much clearer picture of the extent to which occupational carcinogens and mutagens appear as determinants for certain cancers. This expansion would help to prevent and recognize more work-related cancers. It would also make health practitioners more aware of possible occupational causes or aggravating factors for diseases, a key to effective risk mitigation.

For the European Union, PH policy makers need to institute a shared decision-making process involving employees, employers, and OSH representatives to set PH goals to align with OSH goals. This process should include clear target-oriented commitments of each stakeholder for realising the objectives and anticipating means necessary to achieve them. The work includes aligning the legistlation between PH and OSH. Improvement does not necessarily require additional legislation, but it will depend on better integrating and implementing regulations and incorporating the voices of social partners in PH policy. Governments should make full use of existing OSH structures (agencies, providers and services) and empower workplaces to implement them. OSH can work out necessary safety and organizational measures at the company and sectoral levels.

## Moving forward

The COVID-19 pandemic has demonstrated how vital it is that OSH and PH work together effectively. Many workplace health issues are also PH issues and vice versa. Duplication of efforts in respective areas of expertise is inefficient and undesirable; addressing these issues will require thoughtful collaboration to improve governance and strategy formulation within and shared between OSH and PH. Some factors remain unique to the workplace, such as the need for the OSH policy framework to be updated in light of new and emerging risks. The levers to improve OSH include a robust evidence base with reliable, comprehensive, timely data and information. Collaboration and coordination must involve the social partners.

To better protect and improve the health of workers around the world through coordination of public health and occupation safety and health, it will be essential to work with the World Health Organization (WHO) and with the International Labour Organization (ILO). We advocate that worker protection and OSH services as a fundamental right for all workers. An amendment to the ILO Declaration on Fundamental Principles and Rights at Work adopted by the 111th session of the International Labour Conference in 2022 [22, 23], now includes “a safe and healthy working environment” as a fundamental right of workers along with access to occupational health services [24].

The impact of COVID-19 has reaffirmed the importance of well-developed health systems and integration of PH and OSH to protect the health and life of workers, to curb the spread of the disease, and ensure business preparedness and resilience. Strategic collaboration between WHO and ILO could improve planning and implementation of PH and OSH within countries. In February 2022 the WHO-ILO announced commitment to “…strengthen cooperation between the health sector and the institutions of the world to work to address ore effectively the ongoing COVID-19 pandemic, protect all workers from occupational injuries and diseases, ad prepare workplaces for future public health emergencies” [25].

## Conclusions

The European Union needs similar coordination between OSH and PH. The European agency for Occupational Safety and Health could be the lead agency in consultation with the European Centre for Disease Prevention and Control for advice on infection prevention and control and preparedness, public health, and occupational health. Such collaboration was realized to some extent during the pandemic [26]. Despite limited resources there should be national expert OSH agencies, providers and services that can be used by health professionals for specific workplace advice.
